# Exact formula for cubic partitions

**DOI:** 10.1007/s11139-024-00871-0

**Published:** 2024-05-21

**Authors:** Lukas Mauth

**Affiliations:** Division of Mathematics, Department of Mathematics and Computer Science, University of Cologne, Weyertal 86-90, 50931 Cologne, Germany

**Keywords:** Circle Method, $\eta$-function, Partitions, 11B57, 11F03, 11F20, 11F30, 11F37, 11P82

## Abstract

We obtain an exact formula for the cubic partition function and prove a conjecture by Banerjee, Paule, Radu and Zeng.

## Introduction and statement of results

Let *n* be a non-negative integer. A non-increasing finite sequence of positive integers that sums to *n* is called a *partition* of *n*. We denote by *p*(*n*) the number of partitions of *n*,  which can be defined by the coefficients of the following *q*-series [1]

1.1 $$\begin{aligned} \sum _{n=0}^{\infty } p(n)q^n = \prod _{n=1}^{\infty } \frac{1}{1-q^n}. \end{aligned}$$

The function *p*(*n*) is one of the most well-studied functions throughout number theory and satisfies numerous remarkable identities. One example is the following identity discovered by Ramanujan [1, 10]:

$$\begin{aligned} \sum _{n=0}^{\infty } p(5n+4)q^n = 5 \prod _{n=1}^{\infty } \frac{(1-q^{5n})^5}{(1-q^n)^6}. \end{aligned}$$

An immediate consequence is one of Ramanujan’s celebrated congruences which asserts that for any non-negative integer *n*, 

$$\begin{aligned} p(5n+4) \equiv 0 \,\,(\textrm{mod}\,\,{5}). \end{aligned}$$

Similar to (1.1), we define the *cubic partition function* *a*(*n*) by

1.2 $$\begin{aligned} \sum _{n=0}^{\infty } a(n)q^n = \prod _{n=1}^{\infty } \frac{1}{(1-q^n)(1-q^{2n})}. \end{aligned}$$

Chan [6] proved that the function *a*(*n*) satisfies an identity similar to (1.1) above

$$\sum _{n=0}^{\infty } a(3n+2)q^n = 3 \prod _{n=1}^{\infty } \frac{(1-q^3)^3(1-q^6)^3}{(1-q)^4(1-q^2)^4},$$

$$a(3n +2) \equiv 0 \,\,(\textrm{mod}\,\,{3}),$$

see [6]. His result is closely related to Ramanujan’s cubic continued fraction, see [7]. For an introduction to Ramanujan’s cubic continued fraction, see [2, 4]. Furthermore, from the generating function (1.2), it is obvious that *a*(*n*) is the number of partition pairs $(\lambda , \mu )$ such that $|\lambda | + |\mu | = n$ and $\mu$ consists of only even numbers. These connections gave rise to the term “cubic partitions”.

The first 30 values of *a*(*n*) are as follows: 1, 1, 3, 4, 9, 12, 23, 31, 54, 73, 118, 159, 246, 329, 489, 651, 940, 1242, 1751, 2298, 3177, 4142, 5630, 7293, 9776, 12584, 16659, 21320, 27922, 35532, 46092.

There is another combinatorial interpretation of the cubic partition function. Suppose we have a real polynomial equation of degree *n*. Then, *a*(*n*) counts the number of possibilities for the roots to be real and unequal, real and equal (in various combinations), or simple or multiple complex conjugates. For example, for a real cubic polynomial, one can have 3 real distinct roots, 3 distinct real roots, 3 real roots and two of them are equal, 3 equal real roots, and3 real roots and two are equal, 3 real roots, and 1 real root with a complex conjugate pair of 2 roots. Hence, $a(3) = 4$.

Another natural question about *p*(*n*) is how fast it grows. In 1918, Hardy and Ramanujan [8] showed the following with their celebrated Circle Method

1.3 $$\begin{aligned} p(n) \sim \frac{1}{4n\sqrt{3}}\exp \bigg (\pi \sqrt{\frac{2n}{3}}\bigg ), \quad n \rightarrow \infty . \end{aligned}$$

Hardy and Ramanujan made extensive use of the fact that the generating function (1.1) is almost a modular form. More precisely,

1.4 $$\begin{aligned} \sum _{n=0}^{\infty } p(n)q^n = \prod _{n=1}^{\infty } \frac{1}{1-q^n} = \frac{q^{\frac{1}{24}}}{\eta (\tau )}, \end{aligned}$$

where $\eta (z)$ is the Dedekind eta function defined on the upper half-plane $\mathbb {H} = \{\tau \in \mathbb {C}\mid \text {Im}\tau > 0\}$ by

$$\eta (\tau ) = q^{\frac{1}{24}} \prod _{n=1}^{\infty } (1-q^n), \quad q=\exp (2\pi i \tau ).$$

The function $\eta (\tau )$ is a modular form of weight $\frac{1}{2}$ and satisfies the transformation law

$$\begin{aligned} \eta \bigg (\frac{a\tau +b}{c\tau +d}\bigg ) = \varepsilon (a,b,c,d) \sqrt{-i(c\tau + d)}\eta (\tau ), \quad \gamma = \begin{pmatrix} a &{}b \\ c &{} d \end{pmatrix} \in {\text {SL}}_2(\mathbb {Z}), \end{aligned}$$

1.5 $$\begin{aligned} \varepsilon (a,b,c,d) := {\left\{ \begin{array}{ll} e{\frac{\pi i b}{12}} &{} \text {if } c=0, d=1, \\ e^{\pi i \big (\frac{a+d}{12}-s(d,c)\big )} &{} \text {if } c>0. \end{array}\right. } \end{aligned}$$

For the last definition, we used that one can always assume $$c>0$$ or ($$c=0$$ and $$d=1).$$ Here, *s*(*h*, *k*) is the Dedekind sum

$$\begin{aligned} s(h,k) := \sum _{n=1}^{k-1} \frac{n}{k} \bigg (\frac{hn}{k}- \bigg \lfloor \frac{hn}{k}\bigg \rfloor - \frac{1}{2} \bigg ). \end{aligned}$$

A proof can be found in [3]. Later, Rademacher [9] perfected the circle method and obtained the exact formula

1.6 $$\begin{aligned} p(n) = \frac{1}{\pi \sqrt{2}} \sum _{k=1}^{\infty } A_k(n) \sqrt{k}\cdot \frac{d}{dn}\left( \frac{\sinh \bigg [\frac{\pi }{k}\sqrt{\frac{2}{3}\left( n-\frac{1}{24}\right) }\bigg ]}{\sqrt{n-\frac{1}{24}}}\right) , \end{aligned}$$

where $A_k(n)$ is the Kloosterman sum given by

$$\begin{aligned} A_k(n) := \sum _{\begin{array}{c} 0\le m < k \\ \gcd (m,k) = 1 \end{array}} \exp \left( \pi i \left( s(m,k)-\frac{2nm}{k}\right) \right) . \end{aligned}$$

Note that the series (1.6) converges very fast and that the first term recovers the result by Hardy and Ramanujan (1.3). Here, we want to find an exact formula of Rademacher type for the cubic partition function *a*(*n*),  by using a result of Zuckerman [12] extending Rademacher’s work and it can be seen as the apotheosis of the classical Circle Method. Using the Circle Method, Zuckerman computed exact formulas for Fourier coefficients of weakly holomorphic modular forms of arbitrary non-positive weight on subgroups of ${\text {SL}}_2(\mathbb {Z})$ with finite index in terms of the cusps of the underlying subgroup and the negative coefficients of the form at each cusp. We state the relevant results of Zuckerman in Sect. 2 and prove the exact formula for *a*(*n*) in Sect. 3. Similar to the case above, we will obtain as an immediate consequence the asymptotic main term for

$$a(n) \sim \frac{e^{\pi \sqrt{n-\frac{1}{8}}}}{8\left( n-\frac{1}{8}\right) ^{\frac{5}{4}}}, n \rightarrow \infty .$$

Finally, we are going to prove a conjecture by Banerjee, Paule, Radu and Zeng [5] which predicts the following asymptotic formula for $\log a(n)$

$$\begin{aligned}&\log \left( a(n)\right) \sim \pi \sqrt{n} - \frac{5}{4} \log \left( n\right) - \log \left( 8\right) - \left( \frac{15}{8\pi } + \frac{\pi }{16}\right) \frac{1}{\sqrt{n}}, \quad n \rightarrow \infty ,\\&\quad \frac{15}{8\pi } + \frac{\pi }{16} \approx 0.79. \end{aligned}$$

## Zuckerman’s result

The following two sections follow Zuckerman’s work [12] closely. Most of the results are completely taken over and translated into more modern language. His method, due to its generality, involves numerous technical parameters which appear in the Circle Method and do not have obvious meaning without knowing the context they arise in. The interested reader should thus consult [12] for a more detailed account.

Let $\Gamma$ be a subgroup of ${\text {SL}}_2(\mathbb {Z})$ of finite index and let *F* be a weakly holomorphic modular form of weight $k=-r, r>0.$ Thus, $F(\tau )$ satisfies a transformation equation of the form

$$\begin{aligned} F\left( \frac{a\tau + b}{c\tau + d}\right) = \varepsilon (-i(c\tau + d))^kF(\tau ), \quad \begin{pmatrix} a &{}b \\ c &{} d \end{pmatrix} \in \Gamma , \end{aligned}$$

where $\varepsilon = \varepsilon (a,b,c,d)$ lies on the unit circle and depends only on the transformation. If $c\ne 0,$ then *c* is taken to be positive and we choose the branch of the argument such that

$$ -\frac{\pi }{2}< {{\,\textrm{Arg}\,}}(-i(c\tau + d)) < \frac{\pi }{2}.$$

Since $\Gamma$ is of finite index in the modular group, we can choose a complete finite system of representatives for the inequivalent cusps of $\Gamma ,$ which we will denote by $P_1, \ldots P_s,$ where

$$P_g = \frac{p_g}{q_g}, \quad \gcd (p_q,g_q) = 1, \quad g_q > 0, \quad g=1, \ldots , s.$$

In order to treat all our cusps symmetrically, we will have to assume that the point at infinity does not belong to our chosen set of representatives. This can always be achieved by considering a $\Gamma$-equivalent rational point instead. We will obtain a set of Fourier expansions $f_1, \ldots , f_s$ corresponding to our set of cusps in the variable $(\tau - P_g)^{-1}.$ Consider now any transformation $\gamma \in {\text {SL}}_2(\mathbb {Z}),$ which must not necessarily belong to our subgroup $\Gamma .$ We then write

2.1 $$\begin{aligned} F\left( \frac{a\tau + b}{c\tau + d}\right) = \varepsilon ^{*}(-i(c\tau + d))^kF^{*}(\tau ) \end{aligned}$$

where $F^{*}(\tau )$ is now a usual Fourier expansion in $\tau ,$ obtained from one of the expansions

$$f_g(x) = \sum _{m =-\mu _g}^{\infty } a_m^{(g)} x^m \quad x = \exp \left( \frac{-2\pi i}{c_g(\tau - P_g)}\right) ,$$

where $c_g>0$ will be specified below. Note that by choosing the identity matrix in (2.1) we get a usual Fourier expansion $F(\tau ),$ which we want later. Corresponding to each of the cusps $P_g$, we can find a transformation in $\Gamma$

$$\begin{aligned} \tau ' = \frac{a\tau + b}{c\tau + d}, \end{aligned}$$

which can be written in the form

$$\frac{1}{\tau '-P_g} = \frac{1}{\tau - P_g} + c_g, \quad c_g >0.$$

We can recover the transformation in the usual form as

$$\begin{aligned} \tau ' = \frac{(c_gP_g +1) - c_gP_g^2}{c_g\tau + 1 - c_gP_g}. \end{aligned}$$

We then define $\alpha _g$ by the equation $\varepsilon e^{\frac{\pi i}{2}} = e^{-2\pi i \alpha _g},$ where $0\le \alpha _g < 1$ and $\varepsilon$ is taken with respect to $\tau '.$

We now describe how to find $F^{*}(\tau )$ in terms of the Fourier expansions $f_g.$ We therefore choose any transformation $\gamma \in {\text {SL}}_2(\mathbb {Z}).$ Then, $\frac{a}{c}$ is a rational point if $c\ne 0$ or the point at infinity if $c=0.$ In any case, $\frac{a}{c}$ is $\Gamma$-equivalent to exactly one of our cusps $P_g.$ We can therefore find a transformation

$$\frac{a_1P_g + b_1}{c_1P_g + d_1} = \frac{a}{c}, \quad c\ge 0, \quad \begin{pmatrix} a_1 &{}b_1 \\ c_1 &{} d_1 \end{pmatrix} \in \Gamma ,$$

which takes $P_g$ into $\frac{a}{c}.$ We can then express $F^{*}(\tau )$ in terms of the Fourier expansion $f_g$ as follows

$$\begin{aligned} F^{*}(\tau ) = q_g^{k} \exp \left( \frac{2\pi i q_g^2}{c_g} \alpha _g \tau \right) \dot{f}_g\left( \exp \left( \kappa \frac{2\pi i q_g}{c_g} (a_1d-c_1b)\right) \exp \left( \frac{2 \pi i q_g^2}{c_g} \tau \right) \right) , \end{aligned}$$

where $\kappa = \pm 1$ is taken as the solution to the following equations

$$p_q = \kappa (ad_1-cb_1), \quad q_g = \kappa (ca_1-ac_1).$$

Furthermore, one obtains

$$\varepsilon ^*=\varepsilon (a_1,b_1,c_1,d_1) \exp \left( \kappa \frac{\pi i r}{2}\right) \exp \left( \kappa \frac{2\pi i q_g}{c_g} \alpha _g(a_1d-c_1b)\right) .$$

This gives the Fourier expansion in spirit of (2.1) at any rational point $\frac{a}{c},$ including the point at infinity. When executing the circle method with these transformation equations there are numerous parameters that appear. First of all consider as usual the Farey fractions $\frac{h}{k}, \gcd (h,k) = 1, k>0, h\ge 0.$ Then, consider the point $P=P_g - \frac{k}{hc_g}$ which is either a rational point or the point at infinity. In any case, it is $\Gamma$-equivalent to one of our cusps $P_\beta ,$ where $\beta = \beta (h,k,g).$ There exists a transformation in $\Gamma$ which takes this point into $P_\beta$

$$\begin{aligned} \frac{aP+b}{cP+d} = P_\beta , \quad \begin{pmatrix} a &{}b \\ c &{} d \end{pmatrix} \in \Gamma . \end{aligned}$$

Associated to this transformation is the parameter $\sigma _{h,k}^{(g)}$ implicitly defined as the solution of the equations

$$a\left( \frac{c_g}{q_g}p_gh-k\right) + bc_gh = \sigma _{h,k}^{(g)}p_\beta ,$$

$$c\left( \frac{c_g}{q_g}p_gh-k\right) + dc_gh = \sigma _{h,k}^{(g)}q_\beta .$$

It satisfies $-c_g \le \sigma _{h,k}^{(g)} \le c_g, \sigma _{h,k}^{(g)} \ne 0.$ The remaining technical parameters necessary to state Zuckerman’s result are

$$\begin{aligned} \delta _{h,k}^{(g)}= & {} {\left\{ \begin{array}{ll} -1 &{} \text {if } \sigma _{h,k}^{(g)} > 0, \\ 1 &{} \text {if } \sigma _{h,k}^{(g)} < 0, \end{array}\right. } \quad G_{h,k}^{(g)} = - \frac{2\pi }{kc_\beta }\sigma _{h,k}^{(g)}q_\beta (cP_g+d),\\ \Omega _{h,k}= & {} \varepsilon ^{-1}(a,b,c,d) \exp \left( (1-\delta _{h,k})\frac{\pi i r}{2}\right) \\{} & {} \exp \left( -\frac{2\pi i}{k} \left( \alpha _g h + \frac{\sigma _{h,k}^{(g)}q_\beta (cP_g+d)\alpha _\beta }{c\beta }\right) \right) . \end{aligned}$$

Finally, Zuckerman’s result reads now as follows.

### Theorem 2.1

(Zuckerman’s exact formula) Assume the notation and hypotheses above and denote by $I_r$ the Bessel function of order *r*. If $n + \alpha _g > 0,$ then we have

$$\begin{aligned} a_n^{(g)}= & {} \frac{2\pi c_g^{\frac{r-1}{2}}}{(\alpha _g + n)^{\frac{r+1}{2}}} \sum _{k=1}^{\infty }\sum _{\begin{array}{c} 0\le h < k \\ (h,k) = 1 \end{array}} \Omega _{h,k}^{(g)}\exp \bigg (-2\pi i n \frac{h}{k}\bigg )\\{} & {} \times \sum _{\nu = 1}^{\mu _\beta } a_{-\nu }^{\beta } \exp \left( -\nu G_{h,k}^{(g)} i \right) \frac{\left| \sigma _{h,k}^{(g)}\right| q_\beta (\nu -\alpha _\beta )^{\frac{r+1}{2}}}{kc_\beta ^{\frac{r+1}{2}}} I_{r+1}\\{} & {} \left( \frac{4\pi |\sigma _{h,k}^{(g)}|q_\beta \sqrt{(\nu -\alpha _\beta )(\alpha _g + n)}}{k\sqrt{c_gc_\beta }}\right) . \end{aligned}$$

## Exact formula for the cubic partition function

We recall that the cubic partition function was defined by the identity

$$\begin{aligned} \sum _{n=0}^{\infty } a(n)q^n = \prod _{n=1}^{\infty } \frac{1}{(1-q^n)(1-q^{2n})}=q^{\frac{1}{8}}(\eta (\tau )\eta (2\tau ))^{-1}. \end{aligned}$$

Using the relation

$$ 2\frac{a\tau + b}{c\tau + d} = \frac{a(2\tau ) + 2b}{\frac{c}{2}(2\tau ) + d}$$

we can view $\eta (2\tau )$ in light of (1.5) as a modular form of weight $\frac{1}{2}$ on the subgroup $\Gamma _0(2)$ of ${\text {SL}}_2(\mathbb {Z})$ with multiplier

$$\varepsilon = \varepsilon \left( a,2b,\frac{c}{2},d\right) .$$

Hence, we find that

$$F(\tau ) = \frac{1}{\eta (\tau )\eta (2\tau )}$$

is a weakly holomorphic modular form of weight $-1$ with multiplier and thus we can apply Zuckerman’s result to find the Fourier coefficients of $F(\tau )$ and therefore a formula of *a*(*n*). The group $\Gamma _0(2)$ has two inequivalent cusps 0 and $\frac{1}{2}.$ We consider the transformations

$$ \tau ' = \frac{\tau }{2\tau + 1}, \quad \tau ' = \frac{3\tau - 1}{4\tau -1}$$

which we write as

$$\frac{1}{\tau '} = \frac{1}{\tau } + 2, \quad \frac{1}{\tau '-\frac{1}{2}} = \frac{1}{\tau - \frac{1}{2}} + 4.$$

Thus, we find that $c_1 = 2$ and $c_2 = 4.$ Moreover, we can now determine $\alpha _1$ and $\alpha _2.$ Since $F(\tau )$ has the multiplier

$$\varepsilon = \varepsilon (a,b,c,d)^{-1}\varepsilon \left( a,2b,\frac{c}{2},d\right) ^{-1},$$

a short calculation shows that $\alpha _1 = \alpha _2 = \frac{7}{8}.$ Since the point at infinity is $\Gamma _0(2)$-equivalent to $\frac{1}{2},$ we obtain that $F^{*}(\tau )$ is given in terms of the Fourier expansion $f_2.$ To find the exact expression for $F^{*}(\tau )$, we follow Zuckerman’s method by choosing a transformation that maps $\frac{1}{2}$ to the point at infinity. Obviously,

$$ \begin{pmatrix} -1 &{}0 \\ 2 &{} -1 \end{pmatrix} \frac{1}{2} = i\infty .$$

Hence, $$\kappa = -1$$ and

$$\begin{aligned} F^{*}(\tau )=\frac{1}{2}\exp \left( \frac{7\pi i}{8} \tau \right) f_1(-\exp (2\pi i \tau )) = \frac{1}{2}\exp \left( \frac{14\pi i}{8}\right) \sum _{m =-\mu _1}^{\infty } (-1)^m a_m^{(1)}. \end{aligned}$$

Furthermore, we consider the point $\frac{a}{c} = \frac{0}{1},$ which corresponds to the transformation $\tau \mapsto -\frac{1}{\tau }.$ In this case, the obvious equivalent point is 0 itself and the identity is the corresponding transformation. Thus, in this case

$$F^{*}(\tau ) = \exp \left( \frac{7\pi i}{8} \tau \right) f_2(\exp (\pi i \tau )).$$

We now determine $a_{-1}^{(g)}$ and $\mu _{-2}^{(g)}.$ In the first case, we have on the one hand

$$F\left( \begin{pmatrix} 1 &{}0 \\ 0 &{} 1 \end{pmatrix} \tau \right) = F(\tau ) = \varepsilon ^{*}(-1,0,2,-1) \frac{1}{2}\exp \left( \frac{14\pi i}{8}\tau \right) \sum _{m =-\mu _1}^{\infty } (-1)^m a_m^{(1)}$$

with $\varepsilon ^{*} = e^{\frac{7\pi i}{8}}.$ On the other hand, we have by definition that

3.1 $$\begin{aligned} F(\tau ) = e^{-\frac{\pi i \tau }{4}}\left( a(0) + a(1)e^{2\pi i \tau } + \cdots \right) = e^{\frac{2\pi i 7}{8} \tau } \left( a(0)e^{-2\pi i \tau } + a(1) + \cdots \right) \end{aligned}$$

Comparing coefficients we see that $\mu _{-1}^{(2)} = 1$ and $a_{-1}^{(2)} = -2e^{\frac{\pi i}{8}}.$ Furthermore, (3.1) implies that the Fourier coefficients are exactly *a*(*n*) shifted down by one. Thus, we can recover *a*(*n*) through the relation $n=m+1.$ A similar calculation shows that $\mu _{-1}^{(1)} = 1$ and $a_{-1}^{(2)} = \frac{e^{-\frac{\pi i}{2}}}{\sqrt{2}}.$

We now continue to determine the remaining parameters only for $g=2$ as the Fourier expansion at $\frac{1}{2}$ gives us the exact formula. For $\beta (h,k,2)$, it is required to find transformations that send $P_g-\frac{k}{c_gh}$ into $P_\beta .$ Let $h', h'', h'''$ be any solutions of

$$ \left( \frac{k}{2} -h \right) h' \equiv 1 \,\,(\textrm{mod}\,\,{2h}), \quad h' > 0, \quad \text { for } k \equiv 0 \,\,(\textrm{mod}\,\,{4}),$$

$$ kh'' \equiv -1 \,\,(\textrm{mod}\,\,{h}), \quad h'' > 0, \quad \text { for } k \equiv 0 \,\,(\textrm{mod}\,\,{2}),$$

$$ (k-2h)h''' \equiv 1 \,\,(\textrm{mod}\,\,{4h}), \quad h''' > 0, \quad \text { for } k \equiv 1 \,\,(\textrm{mod}\,\,{2}).$$

Then, the following table gives the remaining parameters needed to apply Theorem 2.1:

**Table:** 

*k*		*a*		*b*		*c*		*d*	$\beta$	$\sigma _{h,k}^{(2)}$	$\delta _{h,k}^{(2)}$		$G_{h,k}^{(2)}$
$\equiv 1 \,\,(\textrm{mod}\,\,{2})$		$h'''$		$\frac{(k-2h)h'''-1}{4h}$		$2h''' + 4h$		$\frac{(k-2h)h'''-1}{2h}- 2h + k$	2	$-1$	1		$\left( \frac{h'''k-1}{2kh} + 1\right) \pi$
$\equiv 0 \,\,(\textrm{mod}\,\,{4})$		$h'$		$\frac{\left( \frac{k}{2}-h\right) h'- 1}{2h}$		$2h' + 2h$		$\frac{\left( \frac{k}{2}-h\right) h' - 1}{h} + \frac{k}{2} - h$	2	$-2$	1		$\left( \frac{h'k-2}{kh} + 1\right) \pi$
$\equiv 2 \,\,(\textrm{mod}\,\,{4})$		*h*		$\frac{k-2h}{4}$		$4h''$		$\frac{kh'' + 1}{h} - 2h''$	1	4	$-1$		$-\left( \frac{4h''k+4}{kh}\right) \pi$

Since the series in Theorem 2.1 converges absolutely, the terms can be rearranged and putting everything together, keeping in mind the shift $n = m+1,$ it follows that

3.2 $$\begin{aligned} a(n) =&\frac{e^{-\frac{\pi i}{2}}}{2\sqrt{2}} \sum _{\begin{array}{c} k \equiv 2 \,\,(\textrm{mod}\,\,{4}) \\ k> 0 \end{array}} A_{k}(n+1) \frac{1}{n-\frac{1}{8}} I_2\left( \frac{2\pi \sqrt{n-\frac{1}{8}}}{k}\right) \nonumber \\&- \frac{\pi e^{\frac{\pi i}{8}}}{2} \sum _{\begin{array}{c} k \equiv 0 \,\,(\textrm{mod}\,\,{4}) \\ k> 0 \end{array}} \frac{1}{k} A_k(n+1) \frac{1}{n-\frac{1}{8}} I_2\left( \frac{\sqrt{2}\pi \sqrt{n-\frac{1}{8}}}{k}\right) \nonumber \\&- \frac{\pi e^{\frac{\pi i}{8}}}{4} \sum _{\begin{array}{c} k \equiv 1 \,\,(\textrm{mod}\,\,{2}) \\ k > 0 \end{array}} \frac{1}{k} A_k(n+1) \frac{1}{n-\frac{1}{8}} I_2\left( \frac{\sqrt{2}\pi \sqrt{n-\frac{1}{8}}}{2k}\right) , \end{aligned}$$

where

3.3 $$\begin{aligned} A_k(n)= & {} \sum _{\begin{array}{c} 0\le h < k \\ (h,k) = 1 \end{array}} \varepsilon \left( h, \frac{k-2h}{4}, 4h'', \frac{kh''+1}{h} - 2h'' \right) ^{-1} \exp (\pi i) \nonumber \\{} & {} \times \exp \left( -\frac{2\pi i 7}{8k}h + \frac{1}{8}\left( \frac{2h''k+2}{kh}\right) \pi i - 2\pi in\frac{h}{k}\right) ,\nonumber \\{} & {} k \equiv 2 \,\,(\textrm{mod}\,\,{4}),\end{aligned}$$

3.4 $$\begin{aligned} A_k(n)= & {} \sum _{\begin{array}{c} 0\le h < k \\ (h,k) = 1 \end{array}} \varepsilon \left( h', \frac{\left( \frac{k}{2}-h\right) h' - 1}{2h}, 2h'+2h', \frac{\left( \frac{k}{2} - h\right) h' - 1}{h} + \frac{k}{2} - h \right) ^{-1} \exp (\pi i) \nonumber \\{} & {} \times \exp \left( -\frac{2\pi i 7}{8k}h - \frac{1}{8}\left( \frac{h'k - 2}{kh} + 1\right) \pi i - 2\pi in\frac{h}{k}\right) ,\nonumber \\{} & {} k \equiv 0 \,\,(\textrm{mod}\,\,{4}), \end{aligned}$$

3.5 $$\begin{aligned} A_k(n)= & {} \sum _{\begin{array}{c} 0\le h < k \\ (h,k) = 1 \end{array}} \varepsilon \left( h''', \frac{(k-2h)h''' - 1}{4h}, 2h''' + 4h, \frac{(k-2h)h'''-1}{2h} - 2h + k \right) ^{-1} \exp (\pi i) \nonumber \\{} & {} \times \exp \left( -\frac{2\pi i 7}{8k}h - \frac{1}{8}\left( \frac{h'''k - 1}{2kh} + 1\right) \pi i - 2\pi in\frac{h}{k}\right) ,\nonumber \\{} & {} k \equiv 2 \,\,(\textrm{mod}\,\,{4}), \end{aligned}$$

Changing the order of summation one last time, we arrive at the following

### Theorem 3.1

For $n \ge 1$, we have the exact formula

3.6 $$\begin{aligned}&a(n) = \frac{\pi e^{-\frac{\pi i}{2}}}{4\sqrt{2}\left( n-\frac{1}{8}\right) } \sum _{\begin{array}{c} l \equiv 1 \,\,(\textrm{mod}\,\,{2}) \\ l> 0 \end{array}} \frac{1}{l} A_{l'}(n+1) I_2\left( \frac{\pi \sqrt{n-\frac{1}{8}}}{l}\right) \nonumber \\&- \frac{\pi e^{\frac{\pi i}{8}}}{2\left( n-\frac{1}{8}\right) } \sum _{\begin{array}{c} l \equiv 0 \,\,(\textrm{mod}\,\,{2}) \\ l > 0 \end{array}} \frac{1}{l} A_{l'}(n+1) I_2\left( \frac{\sqrt{2}\pi \sqrt{n-\frac{1}{8}}}{l}\right) , \end{aligned}$$

where $l' = l$ if $l \equiv 1 \,\,(\textrm{mod}\,\,{2}),$ $l' = l$ if $l \equiv 0 \,\,(\textrm{mod}\,\,{4}),$ $l' = \frac{l}{2}$ if $l \equiv 2 \,\,(\textrm{mod}\,\,{4}).$

It is easy to see that the series converges rapidly and that the term $l=1$ dominates the rest of the sum. Thus, we may conclude that

$$a(n) \sim \frac{\pi }{4\sqrt{2}\left( n-\frac{1}{8}\right) } I_2\left( \pi \sqrt{n-\frac{1}{8}}\right) .$$

For the Bessel function of order $\nu \in \mathbb {N}$, the following asymptotics for $z \in \mathbb {R}$ are known,

3.7 $$\begin{aligned} I_\nu (z) \sim \frac{e^{z}}{\sqrt{2\pi z}} \sum _{k=0}^{\infty } (-1)^k \frac{a_k(\nu )}{z^k}, \quad z \rightarrow \infty , \end{aligned}$$

see [11], where $a_k(\nu )$ is defined as

3.8 $$\begin{aligned} a_k(\nu )= & {} (-1)^k\frac{\left( \frac{1}{2} - \nu \right) _k\left( \frac{1}{2} + \nu \right) _k}{2^k k!}, \quad k = 0,1,2,\ldots \end{aligned}$$

3.9 $$\begin{aligned} (\lambda )_n= & {} \frac{\Gamma (\lambda + n)}{\Gamma (\lambda )},\end{aligned}$$

3.10 $$\begin{aligned} (\lambda )_0= & {} 1. \end{aligned}$$

Thus, we find that the first-order approximation is

$$I_2(z) = \frac{e^{z}}{\sqrt{2\pi z}} \left( 1 + O\left( \frac{1}{z}\right) \right) .$$

We now obtain the following asymptotics.

### Corollary 3.2

We have as $n \rightarrow \infty$

3.11 $$\begin{aligned} a(n) \sim \frac{e^{\pi \sqrt{n-\frac{1}{8}}}}{8\left( n-\frac{1}{8}\right) ^{\frac{5}{4}}}. \end{aligned}$$

As a final consequence, we prove a conjecture by Banerjee, Paule, Radu and Zeng [5].

### Corollary 3.3

We have

$$\log a(n) \sim \pi \sqrt{n} - \frac{5}{4} \log n - \log 8 - \left( \frac{15}{8\pi } + \frac{\pi }{16}\right) \frac{1}{\sqrt{n}}, \quad n \rightarrow \infty .$$

### Proof

We need to shift our approximation from $n-\frac{1}{8}$ to *n*. Thus, write

$$\begin{aligned}e^{\pi \sqrt{n-\frac{1}{8}}}= & {} e^{\pi \sqrt{n}}\left( 1-\frac{\pi }{16\sqrt{n}} + O\left( \frac{1}{n}\right) \right) ,\\ \frac{1}{8\left( n-\frac{1}{8}\right) ^{\frac{5}{4}}}= & {} \frac{1}{8n^{\frac{5}{4}}}\left( 1+O\left( \frac{1}{n}\right) \right) . \end{aligned}$$

Together with the second-order approximation for the Bessel function

$$I_2(z) = \frac{e^{z}}{\sqrt{2\pi z}}\left( 1-\frac{15}{8z} + O\left( \frac{1}{z^2}\right) \right) $$

we obtain

$$a(n) = \frac{e^{\pi \sqrt{n}}}{8n^{\frac{5}{4}}}\left( 1-\frac{15}{8\pi \sqrt{n}} + O\left( \frac{1}{n}\right) \right) \left( 1-\frac{\pi }{16\sqrt{n}} + O\left( \frac{1}{n}\right) \right) \left( 1 + O\left( \frac{1}{n}\right) \right) .$$

Taking logarithms, we find as conjectured

$$\begin{aligned} \log a(n) \sim \pi \sqrt{n} - \frac{5}{4} \log n - \log 8 - \left( \frac{15}{8\pi } + \frac{\pi }{16}\right) \frac{1}{\sqrt{n}}, \quad n \rightarrow \infty . \end{aligned}$$

$$\square $$

### Remark

We can refine our proof and obtain stronger asymptotics of the form

$$\begin{aligned} \log a(n) \sim \pi \sqrt{n} - \frac{5}{4} \log n - \log 8 - \left( \frac{15}{8\pi } + \frac{\pi }{16}\right) \frac{1}{\sqrt{n}} + \sum _{j=2}^{\infty } \frac{c_j}{n^{\frac{j}{2}}} \end{aligned}$$

for some real $c_i$ by taking higher order expansions of the involved functions.
